# Supplementary material for: Symptom clusters in chronic kidney disease and their association with people’s ability to perform usual activities
Source: PLoS One. 2022 Mar 2;17(3):e0264312. doi: 10.1371/journal.pone.0264312 (PMC8890635; doi:10.1371/journal.pone.0264312)
Supplement: S2 Table — (DOCX) [file pone.0264312.s002.docx]

**Table S2.** **Sociodemographic characteristics of people on kidney replacement treatments in the UK Renal Registry at 31^st^ December 2016 for the participating centres**

|  | **N** | **Modality (%)** | | | **Male (%)** | **Age, mean (SD)** | **Ethnicity (%)** | | | | **Social deprivation (%)** | | | | | **Time on KRT, mean years (SD)** |
| --- | --- | --- | --- | --- | --- | --- | --- | --- | --- | --- | --- | --- | --- | --- | --- | --- |
|  |  | *HD* | *PD* | *Tx* |  |  | *White* | *Asian* | *Black* | *Other* | *IMD quintile 1 (least deprived)* | *IMD quintile 2* | *IMD quintile 3* | *IMD quintile 4* | *IMD quintile 5 (most deprived)* |  |
| Centre 1 | 2389 | 42.4 | 6 | 51.7 | 58 | 56.9 (15.9) | 59.5 | 26.8 | 10 | 3.7 | 8.3 | 12 | 17.4 | 18.5 | 43.8 | 9.2 (8.3) |
| Centre 2 | 636 | 39.3 | 3.9 | 56.8 | 59.1 | 55 (16.7) | 53.6 | 42.9 | 2.2 | 1.3 | 7.7 | 9.1 | 13.7 | 19.6 | 49.8 | 8.8 (8.3) |
| Centre 3 | 992 | 46 | 6.5 | 47.6 | 63.8 | 60.2 (15.7) | 91.4 | 4.7 | 1.9 | 2.1 | 15.6 | 24.5 | 24.2 | 23.5 | 12.2 | 8.2 (8.2) |
| Centre 4 | 976 | 38.7 | 6.9 | 54.4 | 62.3 | 58.2 (15.8) | 79 | 15.9 | 4.4 | 0.7 | 20.1 | 21 | 23.4 | 18.5 | 17 | 9.5 (8.6) |
| Centre 5 | 542 | 44.5 | 14.2 | 41.3 | 60.1 | 60 (15) | 82.7 | 12 | 2.8 | 2.6 | 18.3 | 19.2 | 20.3 | 19.4 | 22.9 | 7.8 (7.7) |
| Centre 6 | 1110 | 52.6 | 8.2 | 39.2 | 62.3 | 59.9 (14.5) | 47.4 | 12.6 | 35.3 | 4.7 | 10.6 | 13.5 | 18.7 | 28.1 | 29 | 7.2 (7.3) |
| Centre 7 | 3411 | 43.1 | 3 | 53.9 | 60.5 | 59.2 (14.5) | 39.9 | 31.4 | 18.4 | 10.3 | 11.7 | 15 | 25.5 | 30.5 | 17.2 | 8.9 (8) |
| Centre 8 | 1549 | 33.9 | 3 | 63.1 | 60 | 56 (15.5) | 78.9 | 14.6 | 4.8 | 1.7 | 13.5 | 18 | 17.5 | 19.5 | 31.5 | 10.1 (9.1) |
| Centre 9 | 1051 | 30.4 | 5 | 64.6 | 61.7 | 56.9 (15.5) | 92.6 | 4.2 | 1.1 | 2.1 | 15.8 | 16.9 | 16.8 | 24.7 | 25.9 | 10.8 (10) |
| Centre 10 | 1154 | 34.1 | 7.1 | 58.8 | 57.6 | 57.5 (16.5) | 83.3 | 7.5 | 6.1 | 3.1 | 14.8 | 17.5 | 19.3 | 19.3 | 29.2 | 9.9 (9) |
| Centre 11 | 512 | 28.1 | 7.8 | 64.1 | 64.8 | 59.9 (15) | 96.9 | 0.4 | 0.4 | 2.4 | 8.4 | 17.3 | 26.1 | 28 | 20.2 | 9.7 (8.8) |
| Centre 12 | 1423 | 43.1 | 3.9 | 53 | 61.8 | 58.6 (15.8) | 89.4 | 5.1 | 2.4 | 3 | 11.1 | 14.8 | 17.7 | 20.9 | 35.6 | 10.1 (8.9) |
| Centre 13 | 826 | 41.8 | 9.6 | 48.7 | 61.1 | 60.2 (16) | 92.4 | 4.5 | 1.2 | 1.8 | 17.2 | 20.7 | 19.6 | 19 | 23.4 | 9.2 (8.8) |
| Centre 14 | 571 | 55.3 | 12.1 | 32.6 | 64.6 | 60.1 (15.3) | 67.8 | 21.3 | 9.8 | 1.1 | 6.9 | 12.3 | 16 | 19 | 45.9 | 8.2 (7.4) |
| All 14 centres | 17142 | 41.1 | 5.8 | 53.1 | 60.7 | 58.3 (15.5) | 69.1 | 17.4 | 9.4 | 4.1 | 12.5 | 16.1 | 20.2 | 22.9 | 28.3 | 9.2 (8.5) |
| Note. IMD: Index of Multiple Deprivation; KRT: kidney replacement treatments; SD: standard deviation. | | | | | | | | | | | | | | | | |
