# Supplementary material for: Symptom clusters in chronic kidney disease and their association with people’s ability to perform usual activities
Source: PLoS One. 2022 Mar 2;17(3):e0264312. doi: 10.1371/journal.pone.0264312 (PMC8890635; doi:10.1371/journal.pone.0264312)
Supplement: S3 Table — (DOCX) [file pone.0264312.s003.docx]

### Table S3. Baseline characteristics for people with ≥4 missing symptom or without a score for ‘problems with usual activities’ whose questionnaires were excluded from all analyses (values are numbers (% after excluding missing), unless indicated otherwise)

|  | ***All*** | ***Non-RRT*** | ***Peritoneal dialysis*** | ***Haemodialysis*** | ***Transplant*** |
| --- | --- | --- | --- | --- | --- |
| Total n | 282 | 62 (22.0) | 6 (2.1) | 101 (35.8) | 113 (40.1) |
| Gender (male) | 133 (58.3) | 10 (66.7) | 1 (33.3) | 63 (64.3) | 59 (52.7) |
| Missing | 54 | 47 | 3 | 3 | 1 |
| Age (Mean, SD) | 62.9, 14.5 | 68.4, 13.6 | 71.4, 7.1 | 64.9, 15.1 | 57.8, 13.0 |
| Missing | 1 | 1 | 0 | 0 | 0 |
| Ethnicity |  |  |  |  |  |
| White | 196 (83.4) | 18 (85.7) | 4 (100) | 67 (68.4) | 107 (95.5) |
| Asian | 17 (7.2) | 1 (4.8) | 0 (0) | 14 (14.3) | 2 (1.8) |
| Black | 15 (6.4) | 1 (4.8) | 0 (0) | 14 (14.3) | 0 (0) |
| Other | 7 (3.0) | 1 (4.8) | 0 (0) | 3 (3.0) | 3 (2.7) |
| Missing | 47 | 41 | 2 | 3 | 1 |
| Social deprivation ^a)^ |  |  |  |  |  |
| IMD Quintile 1 (least deprived) | 34 (12.2) | 5 (8.6) | 2 (33.3) | 8 (7.9) | 19 (16.8) |
| IMD Quintile 2 | 46 (16.6) | 13 (22.4) | 0 (0) | 9 (8.9) | 24 (21.2) |
| IMD Quintile 3 | 41 (14.8) | 8 (13.8) | 1 (16.7) | 20 (19.8) | 12 (10.6) |
| IMD Quintile 4 | 64 (23.0) | 17 (29.3) | 1 (16.7) | 19 (18.8) | 27 (23.9) |
| IMD Quintile 5 (most deprived) | 93 (33.5) | 15 (25.9) | 2 (33.3) | 45 (44.6) | 31 (27.4) |
| Missing | 4 | 4 | 0 | 0 | 0 |
| Time on KRT in years (Mean, SD) | 9.9, 9.8 | NA | 4.6, 4.7 | 5.1, 6.1 | 14.1, 10.6 |
| Primary Renal Diagnosis |  |  |  |  |  |
| Diabetes | 38 (17.1) | 3 (30) | 2 (100) | 22 (22.4) | 11 (9.8) |
| Glomerulonephritis | 44 (19.8) | 2 (20) | 0 (0) | 12 (12.2) | 30 (26.8) |
| Hypertension | 17 (7.7) | 0 (0) | 0 (0) | 12 (12.2) | 5 (4.5) |
| Polycystic kidney disease | 25 (11.3) | 0 (0) | 0 (0) | 7 (7.1) | 18 (16.1) |
| Pyelonephritis | 17 (7.7) | 1 (10) | 0 (0) | 6 (6.1) | 10 (8.9) |
| Renal vascular disease | 11 (5.0) | 1 (10) | 0 (0) | 9 (9.2) | 1 (0.9) |
| Other | 31 (14.0) | 0 (0) | 0 (0) | 10 (10.2) | 21 (18.8) |
| Uncertain aetiology | 39 (17.6) | 3 (30) | 0 (0) | 20 (20.4) | 16 (14.3) |
| Missing | 60 | 52 | 4 | 3 | 1 |
| Where survey was completed |  |  |  |  |  |
| At home | 36 (20.2) | 5 (10.9) | 2 (40) | 26 (41.3) | 3 (4.7) |
| In clinical settings (outpatient clinic, renal unit, GP practice) | 142 (79.8) | 41 (89.2) | 3 (60) | 37 (58.8) | 61 (95.4) |
| Missing | 104 | 16 | 1 | 38 | 49 |
| How questionnaire was completed |  |  |  |  |  |
| Alone, without support | 127 (74.3) | 28 (62.2) | 3 (60) | 46 (73.0) | 50 (86.2) |
| With support from family member/friend | 37 (21.6) | 16 (35.6) | 2 (40) | 11 (17.5) | 8 (13.8) |
| With support from clinical staff | 7 (4.1) | 1 (2.2) | 0 (0) | 6 (9.5) | 0 (0) |
| Missing | 111 | 17 | 1 | 38 | 55 |
| **Exposure & outcome** |  |  |  |  |  |
| *POS-S Renal* |  |  |  |  |  |
| Number of symptoms (median, IQR) ^c)^ | 6 (3,8) | 5 (3.5,8) | 5.5 (2,9) | 6 (3,9) | 4 (2,7) |
| *EQ-5D-5L* |  |  |  |  |  |
| Problems with usual activities ^d)^ |  |  |  |  |  |
| No problems | 32 (29.4) | 11 (47.8) | 0 (0) | 10 (21.7) | 11 (28.9) |
| Slight problems | 27 (24.8) | 7 (30.4) | 1 (50) | 10 (21.7) | 9 (23.7) |
| Moderate problems | 27 (24.8) | 4 (17.4) | 1 (50) | 12 (26.1) | 10 (26.3) |
| Severe problems | 16 (14.7) | 0 (0) | 0 (0) | 9 (19.6) | 7 (18.4) |
| Unable to do usual activities | 7 (6.4) | 1 (4.3) | 0 (0) | 5 (10.9) | 1 (2.6) |
| Any problems related to ^e)^ |  |  |  |  |  |
| Mobility | 102 (79.7) | 22 (71) | 3 (100) | 45 (88.2) | 32 (74.4) |
| Self-care | 53 (44.5) | 11 (39.3) | 1 (33.3) | 22 (47.8) | 19 (45.2) |
| Pain/discomfort | 91 (75.2) | 21 (75) | 3 (100) | 36 (75.0) | 31 (73.8) |
| Anxiety/depression | 76 (62.8) | 20 (69) | 2 (100) | 33 (67.4) | 21 (51.2) |
| Note. EQ-5D-5L: EuroQOL Five Dimensions - 5 levels version; CKD non-KRT, people with chronic kidney disease not receiving kidney replacement therapy, POS-S Renal: Palliative care Outcome Scale-Symptom Renal, SD: standard deviation. High scores indicate high symptom severity on the POS-S Renal and more problems with the items on the EQ-5D-5L.   1. Based on index of multiple deprivation quintiles ([27](#_ENREF_27)) 2. For people who completed more than one questionnaire, we used the response from their first questionnaire 3. Refers to number of symptoms with a score of >1 (i.e., reports of being at least slightly bothered by a symptom) 4. Domain from the EQ-5D-5L; primary outcome measure of the current study 5. Refers to the remaining four dimensions within the EQ-5D-5L where people scored >1 (i.e., reports of having at least slight problems) | | | | | |
