# Supplementary material for: Symptom clusters in chronic kidney disease and their association with people’s ability to perform usual activities
Source: PLoS One. 2022 Mar 2;17(3):e0264312. doi: 10.1371/journal.pone.0264312 (PMC8890635; doi:10.1371/journal.pone.0264312)
Supplement: S4 Table — (DOCX) [file pone.0264312.s004.docx]

### Table S4. Baseline characteristics for all people with CKD on KRT in the UK Renal Registry at 31^st^ December 2016 (values are numbers (% after excluding missing), unless indicated otherwise)

|  | ***CKD non-KRT*** | ***Peritoneal dialysis*** | ***Haemodialysis*** | ***Transplant*** |
| --- | --- | --- | --- | --- |
| Total n | 17216 | 3581 | 24816 | 33077 |
| Gender (male) | 9406 (54.6) | 2147 (60) | 15262 (61.5) | 20038 (60.6) |
| Age (Mean, SD) | 74.3, 13.6 | 62, 15.9 | 64.9, 15.3 | 53.5, 13.8 |
| Ethnicity |  |  |  |  |
| White | 12076 (90.8) | 2578 (76.8) | 16623 (72.0) | 24917 (79.7) |
| Asian | 837 (6.3) | 414 (12.3) | 3115 (13.5) | 3510 (11.2) |
| Black | 200 (1.5) | 235 (7) | 2464 (10.7) | 1786 (5.7) |
| Other | 184 (1.4) | 130 (3.9) | 888 (3.8) | 1051 (3.4) |
| Missing | 3919 | 224 | 1726 | 1813 |
| Social deprivation ^a)^ |  |  |  |  |
| IMD Quintile 1 (least deprived) | 3867 (22.9) | 571 (17.3) | 2915 (12.7) | 5518 (18.2) |
| IMD Quintile 2 | 3793 (22.4) | 601 (18.2) | 3613 (15.7) | 5905 (19.5) |
| IMD Quintile 3 | 3680 (21.8) | 671 (20.3) | 4418 (19.2) | 6024 (19.9) |
| IMD Quintile 4 | 3173 (18.8) | 709 (21.5) | 5347 (23.3) | 6447 (21.3  ) |
| IMD Quintile 5 (most deprived) | 2397 (14.2) | 747 (22.6) | 6685 (29.1) | 6371 (21.1) |
| Missing | 306 | 282 | 1838 | 2812 |
| Time on KRT in years (mean, SD) | NA | 3, 4.9 | 5.6, 6.7 | 12.4, 9 |
| Primary renal diagnosis |  |  |  |  |
| Diabetes | 0 | 821 (23.4) | 6209 (25.6) | 3592 (11) |
| Glomerulonephritis | 0 | 580 (16.5) | 3585 (14.8) | 7659 (23.4) |
| Hypertension | 0 | 269 (7.7) | 1825 (7.5) | 1747 (5.3) |
| Polycystic kidney disease | 0 | 244 (6.9) | 1431 (5.9) | 4529 (13.8) |
| Pyelonephritis | 0 | 247 (7) | 2037 (8.4) | 4107 (12.6) |
| Renal vascular disease | 0 | 181 (5.2) | 1294 (5.3) | 373 (1.1) |
| Other | 0 | 553 (15.7) | 3859 (15.9) | 5831 (17.8) |
| Uncertain aetiology | 0 | 617 (17.6) | 4060 (16.7) | 4881 (14.9) |
| Missing | 17216 (100) | 69 | 516 | 358 |
| Note. IQR: interquartile range, CKD non-KRT, people with chronic kidney disease not receiving kidney replacement therapy, KRT: kidney replacement therapy, SD: standard deviation. High scores indicate high symptom severity on the POS-S Renal and more problems with the items on the EQ-5D-5L.   1. Based on index of multiple deprivation quintiles ([27](#_ENREF_27)) | | | | |
