# Supplementary material for: Symptom clusters in chronic kidney disease and their association with people’s ability to perform usual activities
Source: PLoS One. 2022 Mar 2;17(3):e0264312. doi: 10.1371/journal.pone.0264312 (PMC8890635; doi:10.1371/journal.pone.0264312)
Supplement: S5 Table — (DOCX) [file pone.0264312.s005.docx]

### Table S5. Baseline characteristics of people with more than one survey (values are numbers (% after excluding missing), unless indicated otherwise)

|  | ***All*** | ***CKD non-KRT*** | ***Peritoneal dialysis*** | ***Haemodialysis*** | ***Transplant*** |
| --- | --- | --- | --- | --- | --- |
| Total n | 699 (100) | 43 (6.15) | 8 (1.14) | 399 (57.08) | 249 (35.62) |
| Gender (male) | 402 (61.1) | 10 (83.3) | 6 (85.7) | 232 (59.5) | 154 (61.8) |
| Missing | 41 | 31 | 1 | 9 | 0 |
| Age (Mean, SD) | 62.38 (15.4) | 68.1 (12.5) | 65.52 (16.3) | 66.96 (13.9) | 53.97 (14.5) |
| Ethnicity |  |  |  |  |  |
| White | 571 (86.3) | 16 (94.1) | 6 (85.7) | 313 (80.5) | 236 (94.8) |
| Asian | 51 (7.7) | 1 (5.9) | 1 (14.3) | 40 (10.3) | 9 (3.6) |
| Black | 30 (4.5) | 0 | 0 | 30 (7.7) | 0 |
| Other | 10 (1.5) | 0 | 0 | 6 (1.5) | 4 (1.6) |
| Missing | 37 | 26 | 1 | 10 | 0 |
| Social deprivation ^a)^ |  |  |  |  |  |
| IMD Quintile 1 (least deprived) | 122 (17.5) | 7 (17.1) | 2 (25) | 67 (16.8) | 46 (18.5) |
| IMD Quintile 2 | 117 (16.8) | 12 (29.3) | 2 (25) | 61 (15.3) | 42 (16.9) |
| IMD Quintile 3 | 115 (16.5) | 5 (12.2) | 0 | 64 (16.0) | 46 (18.5) |
| IMD Quintile 4 | 147 (21.1) | 8 (19.5) | 4 (50) | 75 (18.8) | 60 (24.1) |
| IMD Quintile 5 (most deprived) | 196 (28.1) | 9 (22) | 0 | 132 (33.1) | 55 (22.1) |
| Missing | 2 | 2 | 0 | 0 | 0 |
| Time on KRT at first survey in years (Mean, SD) | 7.43 (8.7) | 0 (0) | 4.26 (5.8) | 5 (6.7) | 12.72 (9.5) |
| Time between surveys in days (Median, IQR) | 203 (133,301) | 210 (91,281) | 256.5 (105,446.5) | 231 (160,318) | 194 (133,246) |
| Primary Renal Diagnosis |  |  |  |  |  |
| Diabetes | 110 (16.9) | 2 (50) | 2 (28.6) | 91 (23.4) | 15 (6.0) |
| Glomerulonephritis | 121 (18.6) | 1 (25) | 2 (28.6) | 63 (16.2) | 55 (22.1) |
| Hypertension | 42 (6.5) | 0 (0) | 0 (0) | 35 (9.0) | 7 (2.8) |
| Polycystic kidney disease | 82 (12.6) | 1 (25) | 0 (0) | 19 (4.9) | 62 (24.9) |
| Pyelonephritis | 76 (11.7) | 0 (0) | 1 (14.3) | 43 (11.1) | 32 (12.9) |
| Renal vascular disease | 24 (3.7) | 0 (0) | 0 (0) | 19 (4.9) | 5 (2.0) |
| Other | 103 (15.9) | 0 (0) | 1 (14.3) | 51 (13.1) | 51 (20.5) |
| Uncertain aetiology | 91 (14.0) | 0 (0) | 1 (14.3) | 68 (17.5) | 22 (8.8) |
| Missing | 50 | 39 | 1 | 10 | 0 |
| **Exposure & outcome** |  |  |  |  |  |
| *POS-S Renal* |  |  |  |  |  |
| Change in number of symptoms |  |  |  |  |  |
| Median (IQR) increase | 2 (1, 4) | 2 (1, 5) | 2 (2, 2) | 2 (1, 4) | 2 (1, 3) |
| Median (IQR) decrease | -2 (-4, -1) | -1 (-2, -1) | -2 (-3, -1) | -3 (-4, -1) | -2 (-4, -1) |
| Stayed the same, n (%) | 147 (21) | 12 (27.9) | 0 | 52 (13) | 83 (33.3) |
| Problems with usual activities |  |  |  |  |  |
| Decreased | 190 (27.2) | 12 (27.9) | 2 (25.0) | 124 (31.1) | 52 (20.9) |
| Stayed the same | 363 (51.9) | 22 (51.2) | 5 (62.5) | 177 (44.4) | 159 (63.9) |
| Increased | 146 (20.9) | 9 (20.9) | 1 (12.5) | 98 (24.6) | 38 (15.3) |
| Note. IQR: interquartile range, CKD non-KRT, people with chronic kidney disease not receiving kidney replacement therapy,, POS-S Renal: Palliative care Outcome Scale-Symptom Renal, KRT: kidney replacement therapy, SD: standard deviation.   1. Based on index of multiple deprivation quintiles ([27](#_ENREF_27)) | | | | | |
