# Supplementary material for: Symptom clusters in chronic kidney disease and their association with people’s ability to perform usual activities
Source: PLoS One. 2022 Mar 2;17(3):e0264312. doi: 10.1371/journal.pone.0264312 (PMC8890635; doi:10.1371/journal.pone.0264312)
Supplement: S6 Table — (DOCX) [file pone.0264312.s006.docx]

### Table S6. Principal component loadings of all symptoms prior to final cluster composition

|  | ***CKD non-KRT*** | | | | ***Peritoneal dialysis*** | | | | | ***Haemodialysis*** | | | | ***Transplant*** | | | |
| --- | --- | --- | --- | --- | --- | --- | --- | --- | --- | --- | --- | --- | --- | --- | --- | --- | --- |
| **Clusters** | Lack of energy & mobility | GI | Mental health | Cron-bach’s α^a^ | Lack of energy & mobility | GI | Mental health | Skin | Cron-bach’s α ^a^ | Lack of energy & mobility | GI | Skin | Cronbach’s α ^a^ | Lack of energy & mobility | GI | Skin | Cron-bach’s α ^a^ |
| **Symptoms** |  |  |  |  |  |  |  |  |  |  |  |  |  |  |  |  |  |
| Pain | **0.52** | 0.11 | 0.19 | 0.80 | 0.36 | -0.05 | **0.62** | -0.12 | 0.71 | **0.66** | 0.03 | 0.02 | 0.84 | **0.72** | 0.1 | -0.02 | 0.87 |
| Shortness of breath | **0.82** | -0.03 | -0.01 | 0.78 | 0.47 | 0.49 | 0.05 | -0.19 |  | **0.61** | 0.05 | 0.07 | 0.84 | **0.75** | 0.09 | -0.08 | 0.87 |
| Weakness | **0.69** | 0.08 | 0.17 | 0.77 | **0.75** | 0.25 | 0.08 | -0.09 | 0.59 | **0.82** | 0.02 | -0.01 | 0.83 | **0.74** | 0.22 | -0.03 | 0.86 |
| Nausea | 0.05 | **0.80** | 0.1 | 0.60 | 0.16 | **0.78** | -0.11 | 0.32 | NA^3^ | 0.15 | **0.85** | -0.03 | 0.47 | 0.1 | **0.88** | -0.05 | 0.58 |
| Vomiting | -0.04 | **0.95** | -0.11 | 0.66 | 0.06 | **0.83** | -0.06 | 0.26 | NA^3^ | -0.02 | **0.94** | -0.02 | 0.52 | -0.1 | **0.98** | 0.03 | 0.65 |
| Poor appetite | 0.25 | **0.61** | -0.04 | 0.65 | 0.32 | 0.5 | 0.3 | -0.15 |  | **0.53** | 0.31 | -0.07 | 0.85 | 0.33 | **0.65** | -0.1 | 0.64 |
| Constipation | 0.28 | **0.58** | -0.18 | 0.70 | † | † | † | † |  | 0.41 | 0.07 | 0.2 |  | 0.47 | -0.08 | 0.3 |  |
| Sore/dry mouth | **0.51** | -0.03 | 0.23 | 0.82 | 0.5 | 0.11 | -0.11 | 0.29 |  | 0.39 | 0.05 | 0.35 |  | **0.62** | 0.03 | 0.14 | 0.88 |
| Drowsiness | **0.74** | 0.16 | -0.09 | 0.78 | **0.73** | 0.01 | 0.02 | 0.08 | 0.65 | **0.7** | 0.07 | 0 | 0.84 | **0.79** | 0.05 | 0 | 0.87 |
| Poor mobility | **0.84** | -0.07 | -0.02 | 0.79 | **0.76** | 0.06 | 0.09 | -0.08 | 0.71 | **0.86** | -0.11 | -0.07 | 0.84 | **0.96** | -0.15 | -0.08 | 0.87 |
| Itching | 0.15 | 0.48 | 0.16 |  | -0.03 | 0.08 | 0.04 | **0.83** | NA^c^ | -0.11 | 0.06 | **0.83** | 0.66 | -0.1 | 0.09 | **0.84** | NA ^c^ |
| Difficulty sleeping | 0.13 | 0.37 | 0.34 |  | 0.01 | 0.02 | 0.41 | 0.51 |  | 0.25 | -0.05 | **0.59** | 0.66 | 0.16 | 0.28 | 0.46 |  |
| Restless legs | 0.35 | 0.14 | 0.31 |  | † | † | † | † |  | 0.25 | -0.1 | **0.63** | 0.65 | 0.4 | 0.02 | 0.45 |  |
| Changes in skin | 0.21 | 0.39 | 0.26 |  | -0.08 | 0.12 | 0.03 | **0.74** | NA^c^ | -0.04 | 0.12 | **0.71** | 0.68 | 0.01 | -0.08 | **0.78** | NA^c^ |
| Diarrhoea | -0.28 | **0.61** | 0.31 | 0.74 | -0.4 | 0.45 | **0.75** | -0.11 | 0.78 | -0.04 | **0.55** | 0.25 | 0.80 | -0.02 | **0.61** | 0.17 | 0.75 |
| Feeling anxious | 0 | -0.04 | **0.89** | NA^c^ | 0.15 | -0.14 | **0.76** | 0.24 | 0.61 | **0.59** | 0.02 | 0.22 | 0.83 | **0.55** | 0.12 | 0.22 | 0.87 |
| Feeling depressed | 0.15 | 0 | **0.79** | NA^c^ | 0.34 | -0.11 | **0.61** | 0.3 | 0.60 | **0.6** | 0.06 | 0.19 | 0.83 | **0.55** | 0.1 | 0.27 | 0.87 |
| Cronbach’s α^b^ | 0.82 | 0.72 | 0.76 |  | 0.74 | 0.85 | 0.74 | 0.56 |  | 0.85 | 0.73 | 0.71 |  | 0.88 | 0.72 | 0.54 |  |
| Note. GI, gastrointestinal; NA, not applicable; CKD non-KRT, people with chronic kidney disease not receiving kidney replacement therapy. **Bold** indicates loadings for variables in the final cluster; standard font indicates that variables were not loaded onto the cluster because they did not have a loading of at least 0.50; underline indicates that variables met the minimum loading criteria but were removed because they cross-loaded on to another cluster with a loading difference of less than 0.20.  † In the Peritoneal dialysis group, constipation and restless legs were excluded from the principal component analysis because they were not related to any other symptoms.  ^a^ Cronbach’s α for the cluster if removing the symptom from the cluster to which it has been finally assigned (indicated in bold). For example, if removing ‘pain’ from the ‘lack of energy & mobility’ cluster in the CKD non-KRT group, the Cronbach’s α for that cluster would decrease from 0.82 to 0.80.  ^b^  Cronbach’s α for the final cluster (loadings of symptoms assigned to that cluster in bold)  ^c^ Not applicable because it requires at least two items required in a cluster to calculate Cronbach’s α | | | | | | | | | | | | | | | | | |
