# Supplementary material for: Symptom clusters in chronic kidney disease and their association with people’s ability to perform usual activities
Source: PLoS One. 2022 Mar 2;17(3):e0264312. doi: 10.1371/journal.pone.0264312 (PMC8890635; doi:10.1371/journal.pone.0264312)
Supplement: S7 Table — (DOCX) [file pone.0264312.s007.docx]

### Table S7. Summary of changes in total symptom cluster scores and changes in usual activities score and per treatment group. Values are numbers (%)

|  | ***All*** | ***CKD non-RRT*** | ***Peritoneal dialysis*** | ***Haemodialysis*** | ***Transplant*** |
| --- | --- | --- | --- | --- | --- |
| **Total n** | 699 (100) | 43 (100) | 8 (100) | 399 (100) | 249 (100) |
| **Change in usual activities score** |  |  |  |  |  |
| -4 | 4 (0.6) | 0 (0) | 0 (0) | 3 (0.8) | 1 (0.4) |
| -3 | 14 (2.0) | 0 (0) | 1 (12.5) | 12 (3.0) | 1 (0.4) |
| -2 | 59 (8.4) | 4 (9.3) | 0 (0) | 42 (10.5) | 13 (5.2) |
| -1 | 113 (16.2) | 8 (18.6) | 1 (12.5) | 67 (16.8) | 37 (14.9) |
| 0 | 363 (51.9) | 22 (51.2) | 5 (62.5) | 177 (44.4) | 159 (63.9) |
| 1 | 106 (15.2) | 6 (14.0) | 0 (0) | 74 (18.5) | 26 (10.4) |
| 2 | 25 (3.6) | 3 (7.0) | 1 (12.5) | 14 (3.5) | 7 (2.8) |
| 3 | 11 (1.6) | 0 (0) | 0 (0) | 7 (1.8) | 4 (1.6) |
| 4 | 4 (0.6) | 0 (0) | 0 (0) | 3 (0.8) | 1 (0.4) |
| **Change in *Lack of energy cluster* score** |  |  |  |  |  |
| -20 to < -15 |  | 0 (0) | 0 (0) | 3 (0.8) | 0 (0) |
| -15 to < -10 |  | 0 (0) | 0 (0) | 11 (2.8) | 0 (0) |
| -10 to < -5 |  | 2 (4.7) | 0 (0) | 48 (12.0) | 21 (8.4) |
| -5 to < 0 |  | 15 (34.9) | 5 (62.5) | 108 (27.1) | 73 (29.3) |
| 0 |  | 8 (18.6) | 0 (0) | 53 (13.3) | 57 (22.9) |
| 0 < to < 5 |  | 14 (32.6) | 3 (37.5) | 122 (30.6) | 71 (28.5) |
| 5 to < 10 |  | 3 (7.0) | 0 (0) | 45 (11.3) | 25 (10.0) |
| 10 to < 15 |  | 1 (2.3) | 0 (0) | 9 (2.3) | 1 (0.4) |
| 15 to 20 |  | 0 (0) | 0 (0) | 0 (0) | 1 (0.4) |
| **Change in *GI cluster* score** |  |  |  |  |  |
| -15 to < -10 |  | 0 (0) | 0 (0) | 1 (0.3) | 0 (0) |
| -10 to < -5 |  | 0 (0) | 1 (12.5) | 3 (0.8) | 2 (0.8) |
| -5 to < 0 |  | 11 (25.6) | 2 (25.0) | 121 (30.3) | 46 (18.5) |
| 0 |  | 13 (30.2) | 4 (50.0) | 171 (42.9) | 136 (54.6) |
| 0 < to < 5 |  | 18 (41.9) | 1 (12.5) | 96 (24.1) | 60 (24.1) |
| 5 to < 10 |  | 1 (2.3) | 0 (0) | 7 (1.8) | 5 (2.0) |
| **Change in *Skin cluster* score** |  |  |  |  |  |
| -15 to < -10 |  |  | 0 (0) | 3 (0.8) | 0 (0) |
| -10 to < -5 |  |  | 0 (0) | 16 (4.0) | 3 (1.2) |
| -5 to < 0 |  |  | 3 (37.5) | 135 (33.8) | 68 (27.3) |
| 0 |  |  | 1 (12.5) | 80 (20.1) | 124 (49.8) |
| 0 < to < 5 |  |  | 4 (50.0) | 142 (35.6) | 52 (20.9) |
| 5 to < 10 |  |  | 0 (0) | 23 (5.8) | 2 (0.8) |
| **Change in *Mental health cluster* score** |  |  |  |  |  |
| -5 to <0 |  | 11 (25.6) | 5 (62.5) |  |  |
| 0 |  | 19 (44.2) | 2 (25.0) |  |  |
| 0< to 5 |  | 13 (30.2) | 1 (12.5) |  |  |
| Note. GI: gastrointestinal, CKD non-KRT, people with chronic kidney disease not receiving kidney replacement therapy. Scores adjusted for age, sex, ethnicity and Index of Multiple Deprivation quintile. | | | | | |
