# Supplementary material for: Symptom clusters in chronic kidney disease and their association with people’s ability to perform usual activities
Source: PLoS One. 2022 Mar 2;17(3):e0264312. doi: 10.1371/journal.pone.0264312 (PMC8890635; doi:10.1371/journal.pone.0264312)
Supplement: Attachment — Submitted filename: Revision.Response to reviewers.docx [file pone.0264312.s008.docx]

**Point-by-point response to reviewers’ comments**

Manuscript: Symptom clusters in chronic kidney disease and their association with patients’ ability to perform usual activities (PONE-D-21-05457)

Page and line numbers refer to those in the ‘clean’ version of the revised manuscript.

Reviewer #1

**1. The abstract, the methodology is not clear and objective and compromises the analysis of the results.**

We have revised the abstract and made several edits with the aim of making our study and methodology clearer.

**2. The background the introduction could focus more on the effects of symptoms and improve the quality of life of these patients.**

In the Introduction, we have clarified that symptoms, and particularly symptom clusters, are associated with poorer quality of life and health functioning and higher levels of depression, and that managing symptoms clusters may improve outcomes related to quality of life. For example, by including sentences such as:

- “*Those with CKD may suffer from 6 to 20 symptoms simultaneously, which negatively affects quality of life and increases the risk of treatment non-adherence, health care utilization, and mortality*.” (page 4, lines 63-65)
- “… previous research proposed clusters related to fatigue, pain, gastrointestinal, and skin symptoms. They also reported negative associations between these clusters and outcomes linked to quality of life, such as health functioning and depression. This implies that managing clusters, rather than individual symptoms, may be an effective way to improve outcomes related to quality of life for people with CKD…” (page 4, lines 75-80)

**3. Methods: This Topic could be more objective with a description of the applied methodology.**

We have further clarified our methodology by making several changes to the Methods section, such as clarifying:

- In the first sentence of the Methods that: “*We conducted secondary analyses of cross-sectional and longitudinal data”* (pg 5, lines 100-101)
- How patient-reported outcome data was collected: (pg 6, lines 116-117; pg 7, lines 143-144) *“People who were interested received a paper copy of the questionnaire, which they returned to their unit upon completion. […] The completed TP-CKD paper-based questionnaires were sent to the UK Renal Registry (UKRR) and scanned into electronic format.”*
- The assessment methods for demographic and treatment data (pg 7, lines 148-150): *“The UKRR annual report describes the definitions and measurement methods for these data items [ref].*”
- Explaining why the self-reported health status question was not included in the analysis (pg 7, lines 136-141): *“We did not consider the remaining EQ-5D-5L items as part of our the outcome measure […] because […] responses could not be scanned into electronic format (self-rated health status).”*
- That we did not perform sensitivity analyses (pg 10, line 213): *“We did not perform any sensitivity analyses.”*

**4. The statistical analyses: there was no robust statistical analysis.**

We have described our approach to the statistical analyses in the Methods section under ‘Secondary data analysis’ (pg 8-10, lines 161-213). This approach is in keeping with previous studies that investigated symptom clusters in people with chronic kidney disease, such as Almutary *et al* 2016 and Thong *et al* 2009. We have referenced these studies in our manuscript. We have also clarified that “*For all analyses, we used SAS version 9.4 for all analyses and considered a p value of <0.5 significant.”* (pg 10, line 212-213).

**5. This study uses data from 2017 and may be more outdated.**

This is a valid point for which we have added the following sentence in the Limitations section of the Discussion to address this (page x, lines x-x):

“*In addition, the data analysed in this study was collected in 2017, and we propose repeating the analysis in the future when updated registry data becomes available*.”

**6. The author could discuss in more detail the follow-up of these patients and opportunities to apply and approach these symptoms.**

In the Discussion, we have suggested to combine our proposed cluster-based approach to symptom assessment with existing clinical practice guidance on symptom management by adding the following sentence (pg 24, lines 361-363):

*“… effective interventions that incorporate our suggested cluster-based approach to symptom assessments, complemented with clinical practice guidance on how to manage symptoms [refs].*”

**7. Is important describe the sociodemographic data of these centers for explain the symptoms is regulary for everybody.**

Thank you for this valuable suggestion. We have added a table presenting the sociodemographic characteristics for all people on a KRT treated by each of the participating centres (see Supplementary material, Table S1).

Reviewer #2

*Title, Abstract, Introduction and Background*

**1. Please do not label patients by a disease or treatment (e.g. CKD patient groups, haemodialysis patient, TP-CKD participants, etc) – amend in the abstract and throughout the manuscript**

We very much appreciate this advice and have addressed this throughout the manuscript as much as possible. For example, we changed ‘kidney patients’ to ‘people with CKD’, and we now state that we ‘categorised the data into treatment groups’, rather than ‘categorising participants into patient groups’.

**2. Please use the current international nomenclature for kidney disease published in the journal Kidney International (Levey et al 2020) – such as kidney replacement therapy.**

We very much appreciate the reviewer pointing us to Levey *et al*’s 2020 nomenclature. We have made considerable changes throughout the text and believe it is now in line with this nomenclature. We have also stated at the start of the Methods that we followed Levey *et al* for reporting our study findings (pg 5, lines 103-106).

**3. No need to use “our”, “we use” “we found”, “our findings” and so on – simply rephrase these types of sentences throughout the manuscript**

We reviewed the manuscript and identified all occasions where we could reasonably remove ‘our’, or replace ‘we’ by a noun without changing the meaning of sentences. Examples in the abstract include:

- ~~“In our analysis,~~ We categorised data by treatment group: …” (Methods section)
- Overall, ~~we identified~~ clusters related to: …” (Results section)

However, in most instances, we used ‘we’ to avoid passive voice, which is in line with all major style guides and advocated by most academic journals. We have therefore kept these phrases as they were.

**4. Add design and data collection methods (symptom tool, etc) into abstract**

Thanks for recommending this edit - we have now stated this in the abstract as follows:

*“We conducted a secondary analysis of both cross-sectional and longitudinal data collected as part of a national service improvement programme in 14 renal centres in England, UK. This data included symptom severity (17 items, POS-Renal) and the extent to which people had problems performing their usual activities (single item, EQ-5D-5L).”*

**5. Please write the abstract and the entire manuscript according to STROBE guidelines.**

We have used the STROBE guideline to review our manuscript and revised it accordingly. For example, we have added information on:

- assessment methods for demographic and treatment data (STROBE item 8):*“The UKRR annual report describes the definitions and measurement methods for these data items [ref].”*
- sensitivity analyses (STROBE item12c): “*We did not perform any sensitivity analyses*.”
- numbers of individuals at each stage of study (STROBE item 13). For example: *“The UKRR received questionnaires from 3,421 people, of whom 282 were excluded because they had ≥4 missing symptom scores or did not complete the ‘usual activities’ item on EQ-5D-5L (see Supplementary material, Table S3 for their characteristics). The remaining 3,139 people who completed at least one TP-CKD questionnaire were included in the analysis.”* (Results pg 10, lines 217-221). Where we did not have this information because of the nature of a secondary data analysis, we further clarified this in the Methods section. For example, “*Centres did not record information on whom they screened for eligibility, who had been confirmed eligible but declined participation, or people’s reasons for declining participation.*” (Methods pg 6, lines 117-119).
- the external validity of our findings (STROBE item 21) (Discussion pg 22, lines 306-309): *“It provides a robust external validation of clusters identified by previous studies, including skin (21, 24, 26, 27), gastrointestinal (20, 22), and mental health (24, 26), which suggests that our findings are generalisable beyond the study context.”*

Lastly, we have stated at the start of the Methods section that we used STROBE to guide the reporting of our study (pg 5, lines 103-106), and included a completed STROBE checklist in the Supplementary material (Table S1) to indicate on which page each STROBE item has been included.

**6. Amend sentence (lines 81-83) as there is at least one study which included patients not receiving KRT (ref 20 - Almutary et al included almost 25% non-KRT [both CKD G4 and G5]).**

We have added the 2016 Almutary *et al* reference to the Introduction and changed the sentence as follows to clarify that most previous studies identified symptom clusters across rather than stratified for treatments/stages (page 5, lines 83-91):

*“CKD symptom clusters have been mainly investigated in people receiving dialysis [refs]. Studies considering other kidney replacement therapies (KRTs) or CKD stages often identified clusters across rather than stratified by treatment or stage [refs incl 2016 Almutary]. … Only few studies looked at symptom clusters specifically for people with a kidney transplant or with CKD but not on KRT (CKD non-KRT)], despite these people reporting a similar symptom burden. This leaves it largely unknown to what extent current clusters generalise across or differ between treatment groups.”*

**7. Almutary et al 2017 also constructed structural equation modelling demonstrating the direct (casual) relationships between symptom clusters and health-related quality of life**

We have added the 2017 Almutary *et al* study as a reference to the following sentence (pg 4, line 75-78): *“In CKD, previous research proposed clusters related to fatigue, pain, gastrointestinal, and skin symptoms. They also reported negative associations between these clusters and patient outcomes linked to quality of life, such as health functioning [refs incl 2017 Almutary] and depression.*”

**8. Sentence (lines 85-86), the phrase “modest sample sizes” is vague – especially when cluster analysis is only possible when there is a sufficient sample size to do this type of statistical analysis.**

We agree that ‘modest sample sizes’ is vague and have further specified this by changing the sentence as follows (pg 5, lines 85-87): *“…; one reason for this lack of stratification might have been the modest sample sizes of <450 people.”*

**9. Arguably more studies on CKD symptom clusters because symptoms are subjective and could reflect particular cultures, health experiences, etc, etc. There is no need to avoid needing replication studies in another patient population to add to evidence.**

To better explain the rationale for our study, we have amended the following paragraph in the Introduction as follows (page 5, lines 83-91):

*“CKD symptom clusters have been mainly investigated in people receiving dialysis [refs]. Studies considering other kidney replacement therapies (KRTs) or CKD stages often identified clusters across rather than stratified by treatment or stage [refs]; one reason for this lack of stratification might have been the modest sample sizes of <450 people [refs]. Only few studies looked at symptom clusters specifically for people with a kidney transplant [refs] or with CKD but not on KRT (CKD non-KRT) [ref], despite them reporting a similar symptom burden [refs]. This leaves it largely unknown to what extent current clusters generalise across or differ between treatment groups.”*

**10. Clarify the aims related to longitudinal data collection.**

Thank you for this helpful suggestion. We have clarified the aim of the longitudinal analyses in the the introduction as follows (pg 5, lines 92-95):

*“This study, therefore, aimed to (1) explore symptom clustering in a large data set, stratified by CKD treatment group,(2) assess the relevance of the identified clusters by investigating their association with people’s ability to perform their usual activities, and (3) determine if these associations were stable over time.”*

*Methods*

**11. Ensure all STROBE aspects are explicitly described.**

See our response to reviewer 2 point 5

**12. For the TP-CKD program, at grade of CKD are people enrolled? If CKD G4 were included, then further clarity in data analysis and results will be required; that is, how were the different groups handled (i.e. CKD G4, CKD G5, KRT groups)**

The data set did not include information on CKD stage at enrollment in the non-KRT group. We have clarified this in footnote a) to Table 1 as follows:

“*[the CKD non-KRT treatment group] included people with any stage of CKD not on KRT who were under the treatment of a kidney centre. The dataset did not contain information on the stage of CKD at enrollment”*

**13. Clarify how PROM data was collected.**

We have clarified this by adding/changing the following sentences to the Methods (pg 6, lines 116-117; pg 7, lines 143-144): *“People who were interested received a paper copy of the questionnaire, which they returned to their unit upon completion. [..] The completed TP-CKD paper-based questionnaires were sent to the UK Renal Registry (UKRR) and scanned into electronic format.”*

**14. Clarify ethical procedures for TP-CKD.**

We have clarified that no formal ethical approval was required for TP-CKD (pg 7-8, lines 151-160): “*Because the primary purpose of TP-CKD was service evaluation and improvement rather than research, no formal ethical approval was required. People taking part in the TP-CKD programme implicitly consented to their data being processed and linked by returning a completed questionnaire to their kidney centre. The UKRR holds permissions under s251 of the National Health Service (NHS) Act 2006, to gather, process, and share confidential patient information for the purposes of audit and research. These permissions are renewed annually by the UK’s Health Research Authority’s Confidentiality Advisory Group. The collection and secondary analysis of the data for this study were approved by the UKRR (ref: UKRR ILD32) and carried out under the ethical permissions granted to the UKRR by the Research Ethics Committee.*”

**15. Data analysis – at what level was significance set?**

Thank you for noting this. We have added the following sentence to the end of the Methods section (pg 10, lines 212-213): “*For all analyses, we used SAS version 9.4 for all analyses and considered a p value of <0.5 significant.”*

*Discussion*

**16. First paragraph – there is no need to restate design, sample and sites for the study, instead interpret the meaning of the overall results (without restating actual numbers).**

We have removed the first sentence stating the design, sample and sites, and in line with STROBE, focused the first paragraph on a summary of findings for each of the study objectives (pg 22, lines 299-303): *“This study found that, overall, CKD symptom clusters related to lack of energy and mobility, gastrointestinal, skin, and mental health. Although clusters varied between treatment groups, the ‘lack of energy and mobility’ clusters were consistently associated with having problems doing usual activities across groups, both cross-sectionally and longitudinally.”*

**17. Add to several paragraphs in the discussion what explanation can be made for the differences found between this study and previous studies on symptom clusters (why isn’t there more consistency which is only partly explained by different symptom measures used). C. Miaskowski research in cancer symptom clusters and H.A. de Von in CVD symptom clusters is likely to be insightful for this manuscript.**

Thank you for pointing us to the highly relevant paper by Miaskowski *et al*. We have referenced this work in the Discussion’s ‘Relation to other studies’ section as follows:

- (pg 22, lines 305-311) *“We used the largest data set to date to investigate symptom clusters across treatment groups in people with CKD. It provides a robust external validation of clusters across treatment groups identified by most previous studies, including skin, gastrointestinal, and mental health […]. This alignment with previous research on CKD symptom clusters is in contrast to other disease areas, such as cancer, where there seems to be less consistency in symptom clusters across studies [ref to Miaskowski].”*
- (pg 22-23, lines 318-322): *“Furthermore, the ‘uraemic’ cluster in other studies included nausea and poor appetite (20, 21, 26, 27), whereas in our study, these symptoms, together with vomiting, formed a gastrointestinal cluster. This finding strongly aligns with wider symptom cluster research commonly identifying a separate gastrointestinal cluster consisting of nausea and vomiting [ref to Miaskowski].”*

We also provided an explanation for the differences between our findings and those of some others with regards to mental health as a separate cluster (pg 23, lines 333-339):

*“In the CKD non-KRT treatment group, we found that frequently co-occurring mental health symptoms, i.e. feeling anxious and feeling depressed, formed a stand-alone cluster, which differs from what Lee and Jeon reported in their smaller, Korean study. An explanation for mental health being a separate cluster may be that, as people approach kidney failure, they often experience more psychological symptoms. In the peritoneal dialysis group in the current study, this cluster also included pain and diarrhoea, both of which may negatively affect mental health.”*

**18. Lack of energy and its similarity (or difference) to fatigue also requires further interpretation.**

Thank you for this constructive suggestion. We have added the following section to the Discussion to address this (pg 23, lines 325-332):

“*The POS-S Renal also does not include fatigue, which is a common symptom in people with CKD that others found to pertain to several symptom clusters. Lack of energy and weakness represent some but not all aspects of fatigue, which is a multi-faceted symptom related to sociodemographic, psychological, clinical and biochemical factors. This implies that part of the people reporting lack of energy and weakness in our study might have actually suffered from fatigue, which may partly explain the strong associations we found between ‘lack of energy and weakness’ clusters and people’s ability to do usual activities*.”

**19. In the discussion on this study’s symptoms and activity, it would also be worth reviewing and discussing with regard to Almutary et al 2017 CKD symptom cluster modelling which demonstrated significant casual relationships with physical function (and also with mental health)**

We agree this is a relevant study to reference in this context. We have added it to the following statement in the Discussion (pg 23, line 340-343):

*“Our analyses showed that the ‘lack of energy and mobility’ clusters were associated with problems performing usual activities in all groups and models, which echoes the findings of previous research in which uraemic clusters were associated with impaired physical and mental functioning [refs incl Almutary et al 2017].”*

*Clinical Implications*

**20. Clarify why the IPOS-Renal is not recommend as this PROM is widely used (rather than POS-S renal).**

The two main reasons for using the POS-S Renal instead of the IPOS-Renal are:

1. The POS-S Renal contains 17 common CKD symptoms to use for composing clusters, while the IPOS-Renal only includes a subset of these symptoms.
2. The IPOS-Renal includes additional items on concerns beyond symptoms, such as information needs and practical issues. These items would not have been included in the symptom cluster analysis.

We propose not to include this explanation in the manuscript because there is a risk it may cause confusion rather than provide clarity.

*Future Research*

**21. C. Miaskowski and colleagues are exploring underlying mechanisms in cancer symptoms and symptom cluster. There is an opportunity for researchers in CKD to draw from this work and extend it.**

Thank you for this helpful suggestion. We have added a more explicit reference to Miaskowski’s paper in the Discussion as follows (pg 27, lines 419-422):

*“Some have suggested that certain symptoms may share an underlying mechanism or one symptom that ‘drives’ them. Future studies, building on and adding to the wider cluster research agenda [ref to Miaskowski], are warranted to enhance our understanding in this area, alongside qualitative studies to ascertain the clinical face validity of symptom clusters.”*

Reviewer #3

**1. Currently, the terminology of CKD has been revised, enabling researchers to compare results across studies conveniently. For example, end-stage kidney failure or end-stage kidney disease, now termed as kidney failure. Please refer. Levey et al. (2020). Therefore, it is recommend to reword CKD terminology throughout the manuscript to fit current guidelines.**

We very much appreciate this advice and pointing us to the Levey *et al* 2020 nomenclature. We have made considerable changes throughout the text and believe it is now in line with this nomenclature. We have also stated at the start of the Methods that we followed Levey et al for reporting our study findings (pg 5, lines 103-106).

**2. It is also suggested not to label people living with chronic diseases as patients (e.g., line 89: CKD patient groups) consistently throughout the manuscript.**

We very much appreciate this advice and have addressed this throughout the manuscript as much as possible. For example, we changed ‘kidney patients’ to ‘people with CKD’, and we now state that we ‘categorised the data into treatment groups’, rather than ‘categorising participants into patient groups’.

**3. Methods: Line 119: Spell out EQ5D5L first, before using it in abbreviated form.**

Thank you for spotting this – we have now spelled it out on first mention as follows (pg 7, line 131-132):

*“As the outcome measure we used an item from the EuroQol 5 dimensions - 5 level (EQ-5D-5L) version, which was also included in the TP-CKD questionnaire.”*

**4. Methods: Line 128: It is not clear that what do you mean by ‘see below’ as there is no further information given in relation to self-rated health.**

We agree that this was not very clear in the text. We have removed the ‘see below’ reference and instead explained why the self-reported health status questions was not included in the analysis (pg 7, lines 136-141):

*“We did not consider the remaining EQ-5D-5L items as part of our the outcome measure [..] because [… responses could not be scanned into electronic format (self-rated health status).”*

**5. Under discussion, relation to other studies, second paragraph; it is better to mention frequently co-occurring mental health symptoms. i.e., anxiety and depression**

We have taken your suggestion on board and reworded this sentence as follows (pg 23, lines333-334):

*“In the CKD non-KRT treatment group, we found that frequently co-occurring mental health symptoms, i.e. feeling anxious and feeling depressed, formed a stand-alone cluster, …”*

**6. According to current evidence, fatigue is the most frequent symptom experienced by those with CKD. See for example, Almutary et al. (2013) and (2016) in your reference list. However, POS-S-renal doesn’t capture fatigue. It is note that weakness/lack of energy is different to fatigue. Therefore, it is important to discuss this point in the discussion under the section ‘relation to other studies’.**

Thank you for this constructive suggestion. We have added the following sentences to the Discussion Section ‘Relation to Other Studies’ to address this (pg 23, lines 325-332):

“*The POS-S Renal also does not include fatigue, which is a common symptom in people with CKD that others found to pertain to several symptom clusters. Lack of energy and weakness represent some but not all aspects of fatigue, which is a multi-faceted symptom related to sociodemographic, psychological, clinical and biochemical factors. This implies that part of the people reporting lack of energy and weakness in our study might have actually suffered from fatigue, which may partly explain the strong associations we found between ‘lack of energy and weakness’ clusters and people’s ability to do usual activities*.”

**7. Use the RRT abbreviation consistently throughout the manuscript. Abbreviated form of renal replacement therapy is missing in the conclusions section.**

Thank you for spotting this. We have changed renal replacement therapy to KRT in line with rest of the manuscript.

**8. It is suggested to check the reference list to align journal requirements.**

We have corrected instances where references were not in line with journal requirements.
