# Supplementary material for: Symptom clusters in chronic kidney disease and their association with people’s ability to perform usual activities
Source: PLoS One. 2022 Mar 2;17(3):e0264312. doi: 10.1371/journal.pone.0264312 (PMC8890635; doi:10.1371/journal.pone.0264312)
Supplement: Attachment — Submitted filename: Response to reviewers.22 10 21.docx [file pone.0264312.s009.docx]

# Point-by-point response to reviewers’ comments

Manuscript: Symptom clusters in chronic kidney disease and their association with patients’ ability to perform usual activities (PONE-D-21-05457)

Page and line numbers refer to those in the ‘clean’ version of the revised manuscript.

# Reviewer #1:

**Dear author, I recommend reviewing the abstract needs to include that I work with adults.**

Thank you for your suggestion. We have made it clear in the abstract (Line 29) that the data presented in the manuscript is with adults. In the Methods (Line 118), it is stated that people were eligible if they were ‘18 years or over’.

**For methods you need We have clarified that no formal ethical approval was required for TP-CKD and the follow-up period.**

In our previous Response to Reviewers, we revised the ethics section of the Methods to directly address this (see below - Lines 151-160). No formal ethical approval is required in the UK for service evaluation and improvement, regardless if it carried out in a cross-sectionally or longitudinally (like the TP-CKD).

We have revised Line 154 to indicate that no formal ethical approval was required if people completed more than one questionnaire:

“Because the primary purpose of TP-CKD was service evaluation and improvement rather than research, no formal ethical approval was required. People taking part in the TP-CKD programme implicitly consented to their data being processed and linked by returning a completed questionnaire**(s)** to their kidney centre.” Lines 151-154

**Do you need to include more information about the inclusion and exclusion criteria (age, therapy, literate, other condition).**

The full inclusion and exclusion criteria are listed in Lines 112-113: People were eligible if they were aged 18 years or over and receiving care for any stage of CKD or on any form of KRT.

To confirm, there were no other inclusion and exclusion criteria for TP-CKD.

**Describe in the methodology how the questionnaires were applied.**

Key methods for the questionnaires are described in lines 100-141 of the manuscript:

We conducted a secondary analyses of cross-sectional and longitudinal data collected in the context of a national service improvement programme in 14 kidney centres across England (UK) called *Transforming Participation in Chronic Kidney Disease* (TP-CKD) [31]. We followed the Strengthening the Reporting of Observational Studies in Epidemiology (STROBE) guidelines [32] (see Supplementary material, Table S1) and the nomenclature for kidney function and disease proposed by Levey and colleagues [33] for reporting our findings.

## Transforming Participation in Chronic Kidney Disease (TP-CKD)

The TP-CKD programme aimed to support people with CKD to better manage and make decisions about their own care and treatment. This included introducing collection of patient-reported outcome data in 14 English kidney centres [34]. Between December 2015 and December 2017, members of local kidney care teams approached eligible patients in their centre. People were eligible if they were aged 18 years or over and receiving care for any stage of CKD or on any form of KRT. The number and sociodemographic characteristics of people on KRT treated in each centre are provided in Supplementary material, Table S2; this does not include people with CKD not on KRT because this data was not available. People who were interested received a paper copy of the questionnaire, which they returned to their unit upon completion. Centres did not record information on whom they screened for eligibility, who had been confirmed eligible but declined participation, or on reasons for declining participation. In twelve centres, people who had previously taken part were invited to complete the questionnaire again at a later time.

## Measures of exposure and outcome

In the current secondary analysis, we used symptom burden as the measure of exposure. To assess exposure, we analysed data collected through the Palliative care Outcome Scale-Symptom (POS-S) Renal [35], which was one of the patient-reported outcome measures included in the paper-based TP-CKD questionnaire. The POS-S Renal consists of 17 symptoms that are common for people with CKD [35], such as pain, weakness or lack of energy, and itching. For each symptom, respondents are asked to indicate to what extent they have been bothered by it over the last week on a scale from 0 (not at all) to 4 (overwhelmingly).

As the outcome measure we used an item from the EuroQol 5 dimensions - 5 level (EQ-5D-5L) version [36], which was also included in the TP-CKD questionnaire. The EQ-5D-5L assesses general health status and asks respondents to rate whether they had problems doing their usual activities (e.g. work, study, housework, leisure activities) on a scale from 1 (no problem) to 5 (unable). People with CKD previously reported this item as being important to ‘living well’ (29). We did not consider the remaining EQ-5D-5L items as part of the outcome measure because: some fully overlapped with symptoms in the POS-S Renal (pain/discomfort; anxiety/depression; mobility); one was not reported by people with CKD as a priority outcome in research (self-care: wash and dress) [37,38]; and one because its responses could not be scanned into electronic format (self-rated health status).

**13. Clarify how PROM data was collected, this topic needs to be clear.
this topic is still unclear:**

PROM data were collected as part of the TP-CKD survey which is described in lines 100-141 of the manuscript:

We conducted a secondary analyses of cross-sectional and longitudinal data collected in the context of a national service improvement programme in 14 kidney centres across England (UK) called *Transforming Participation in Chronic Kidney Disease* (TP-CKD) [31]. We followed the Strengthening the Reporting of Observational Studies in Epidemiology (STROBE) guidelines [32] (see Supplementary material, Table S1) and the nomenclature for kidney function and disease proposed by Levey and colleagues [33] for reporting our findings.

## Transforming Participation in Chronic Kidney Disease (TP-CKD)

The TP-CKD programme aimed to support people with CKD to better manage and make decisions about their own care and treatment. This included introducing collection of patient-reported outcome data in 14 English kidney centres [34]. Between December 2015 and December 2017, members of local kidney care teams approached eligible patients in their centre. People were eligible if they were aged 18 years or over and receiving care for any stage of CKD or on any form of KRT. The number and sociodemographic characteristics of people on KRT treated in each centre are provided in Supplementary material, Table S2; this does not include people with CKD not on KRT because this data was not available. People who were interested received a paper copy of the questionnaire, which they returned to their unit upon completion. Centres did not record information on whom they screened for eligibility, who had been confirmed eligible but declined participation, or on reasons for declining participation. In twelve centres, people who had previously taken part were invited to complete the questionnaire again at a later time.

## Measures of exposure and outcome

In the current secondary analysis, we used symptom burden as the measure of exposure. To assess exposure, we analysed data collected through the Palliative care Outcome Scale-Symptom (POS-S) Renal [35], which was one of the patient-reported outcome measures included in the paper-based TP-CKD questionnaire. The POS-S Renal consists of 17 symptoms that are common for people with CKD [35], such as pain, weakness or lack of energy, and itching. For each symptom, respondents are asked to indicate to what extent they have been bothered by it over the last week on a scale from 0 (not at all) to 4 (overwhelmingly).

As the outcome measure we used an item from the EuroQol 5 dimensions - 5 level (EQ-5D-5L) version [36], which was also included in the TP-CKD questionnaire. The EQ-5D-5L assesses general health status and asks respondents to rate whether they had problems doing their usual activities (e.g. work, study, housework, leisure activities) on a scale from 1 (no problem) to 5 (unable). People with CKD previously reported this item as being important to ‘living well’ (29). We did not consider the remaining EQ-5D-5L items as part of the outcome measure because: some fully overlapped with symptoms in the POS-S Renal (pain/discomfort; anxiety/depression; mobility); one was not reported by people with CKD as a priority outcome in research (self-care: wash and dress) [37,38]; and one because its responses could not be scanned into electronic format (self-rated health status).

**10. Clarify the aims related to longitudinal data collection.**

We addressed this by explicitly stating the aims of the longitudinal analysis in lines 92-97 of the Introduction:

“This study, therefore, aimed to (1) explore symptom clustering in a large data set,

stratified by CKD treatment group,(2) assess the relevance of the identified clusters by

investigating their association with people’s ability to perform their usual activities, and

**(3) determine if these associations were stable over time**.” Lines 92-97

**In the discussion, check whether the treatment time also interferes with symptoms.**

In this study we aimed to explore symptom clusters and the association between these clusters and the ability to perform usual activities. We described time on KRT for the participants, and adjusted for time on KRT when exploring the association between symptoms and ability to perform usual activities. The effect of time on KRT on symptom burden was not an aim of this work so we have not included it in the discussion.

# Reviewer #2:

No further comments to address.

Thank you, Dr. Bonner, for your constructive feedback on the manuscript.

# Reviewer #3:

No further comments to address.

We appreciate your constructive feedback and kind words on the manuscript, Dr. Yapa.
