# Supplementary material for: Symptom clusters in chronic kidney disease and their association with people’s ability to perform usual activities
Source: PLoS One. 2022 Mar 2;17(3):e0264312. doi: 10.1371/journal.pone.0264312 (PMC8890635; doi:10.1371/journal.pone.0264312)
Supplement: Attachment — Submitted filename: Response to reviewers 13 12 21.docx [file pone.0264312.s010.docx]

# Point-by-point response to reviewers’ comments

Manuscript: Symptom clusters in chronic kidney disease and their association with patients’ ability to perform usual activities (PONE-D-21-05457R2)

Note: Page and line numbers refer to those in the ‘clean’ version of the manuscript.

Reviewer #1

1. **This manuscript is a cross-sectional analysis of patients undergoing renal treatment. In the Transforming Participation in Chronic Kidney Disease (TP-CKD) session, better describe the program, how were the patients asked to fill out and is there an interval for re-application of the questionnaires? Such missing information compromises the analysis of table 3.**

In response to reviewer comments on previous versions of the manuscript, we endeavoured to provide the necessary background information on the TP-CKD programme in a dedicated section in the Methods (Ln 107-121):

*“The TP-CKD programme aimed to support people with CKD to better manage and make decisions about their own care and treatment. This included introducing collection of patient-reported outcome data in 14 English kidney centres [34]. Between December 2015 and December 2017, members of local kidney care teams approached eligible patients in their centre. People were eligible if they were aged 18 years or over and receiving care for any stage of CKD or on any form of KRT. The number and sociodemographic characteristics of people on KRT treated in each centre are provided in Supplementary material, Table S2; this does not include people with CKD not on KRT because this data was not available. People who were interested received a paper copy of the questionnaire, which they returned to their unit upon completion. Centres did not record information on whom they screened for eligibility, who had been confirmed eligible but declined participation, or on reasons for declining participation. In twelve centres, people who had previously taken part were invited to complete the questionnaire again at a later time.”*

2. **Another important data for table 3 is to include the time between analyses. Several studies point to the impact of treatment time and outcomes of interest.**

Table 3 (p. 20) presents the results of the cross-sectional analysis of the data, and as such, there is no time element for this part of our analyses. For the longitudinal analysis (presented in Table 4, p. 21), we reported the time between measurements in the text (Ln 234-5): *“A subsample of 699 people completed follow-up questionnaires at a median of 203 days (IQR, 133 to 301) after baseline, while remaining in the same treatment group.”*

3. **Data on frequency, initiation of this analysis and inclusion of this questionnaire in renal care services are not described. Is there a protocol for applying the questionnaire annually?**

In the Methods section (Ln 107-121; also see response to point 1), we provided details of the TP-CKD programme with regard to when the data was collected, in whom and how. As described in the Methods, the programme ran between December 2015 and December 2017 (Ln 110-111). We this we implied there is no protocol for applying the questionnaire annually.

4. **lines 117 to 121: “Interested people received a printed copy of the questionnaire, which they returned to the unit after completing it. Sites did not record information about who they screened for eligibility, who was confirmed eligible but refused participation, or about the reasons for denial. In twelve centers, people who had already participated were invited to answer the questionnaire later.” It is important to inform the interval between responses for the analysis of table 3. The symptoms improved, which may be a reflection of the treatment and others not, such finding may be due to the treatment process itself. Such observations should be placed as limitations, as well as the non-homogeneous adherence of the centers.**

Table 3 (p. 20) presents the results of the cross-sectional analysis of the data, and as such, there is no time element for this part of our analyses. For the longitudinal analysis (presented in Table 4, p. 21), we reported the time between measurements in the text (Ln 234-5): *“A subsample of 699 people completed follow-up questionnaires at a median of 203 days (IQR, 133 to 301) after baseline, while remaining in the same treatment group.”*

In relation to the symptoms having improved, we would like to clarify that the aim of our study was to identify symptom clusters and their relationship to people’s performing daily activities, both cross-sectionally and longitudinally. The aim was not to describe symptom trajectories over time and as such, we do not report whether symptoms improved in the main text of the manuscript. As background information to the longitudinal analysis, we did include information in the supplement (Table S7) on how symptoms and people’s ability to perform daily activities changed over time. However, this information suggests that symptoms only improved over time for approximately half of patients, while they deteriorated for the other half. We, therefore, do not think these observations indicate clear limitations or ‘non-homogeneous adherence of centres’.

5. **In the discussion, the main symptom identified 'lack of energy and mobility' may be related to the treatment time and its impact, which must be considered, long therapies, displacements...**

To address the impact of treatment time, we have included ‘time on kidney replacement therapy’ in the partially and fully adjusted models of all analyses (see Table 3 (p. 20) and Table 4 (p. 21) to account for the influence this factor can have on symptoms.
